# Nephron-Sparing Surgery for Adenocarcinoma in a Renal Allograft

**DOI:** 10.1155/2012/692986

**Published:** 2012-07-17

**Authors:** Fernando Vázquez Alonso, Enrique Cardozo Rodríguez, Ignacio Puche Sanz, Jose Francisco Flores Martin, Jose Miguel Molina Hernandez, Raquel Berrio Campos, Javier Vicente Prados, Antonio Medina Benitez, Jose Manuel Cózar Olmo

**Affiliations:** ^1^Department of Urology, Hospital Universitario Virgen de las Nieves, Avenida de las Fuerzas Armadas, 18014 Granada, Spain; ^2^Department Radiology, Hospital Universitario Virgen de las Nieves, Avenida de las Fuerzas Armadas, 18014 Granada, Spain

## Abstract

The incidence of malignant tumors in recipients of renal allografts is higher than in the general population. Renal cell carcinoma (RCC) accounts for 4.6% of the tumors in transplanted patients; of them, only 10% are found in transplanted kidneys. Transplantectomy has always been the usual treatment. However, during the last years, nephron-sparing surgery of the allograft is more frequently done in well-selected cases, and therefore dialysis can be avoided. We report the case of a 37-year-old female patient with renal transplant, diagnosed with a 4.5 cm tumor in the lower pole of the renal allograft. The patient underwent partial nephrectomy successfully. Six years after surgery, there is no evidence of recurrence of the disease and the patient maintains an adequate renal function.

## 1. Introduction

The incidence of renal cell carcinoma (RCC) is higher in patients with chronic renal failure treated with dialysis and in kidney transplant patients. Immunosuppression makes transplanted patients more vulnerable to tumors than the general population. Owing to this fact, native kidneys in these patients should be controlled annually by sonography [1].

## 2. Case Presentation

**Figure 1:**
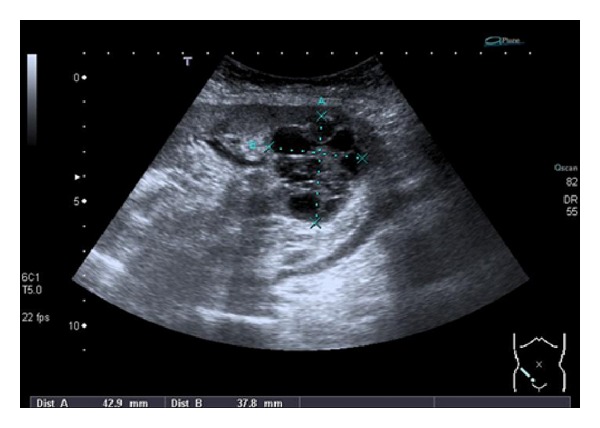
Echo sonography showing a 4.3 × 3.7 cm multilocular cystic mass in the lower pole of the renal allograph suggesting renal cell carcinoma.

**Figure 2:**
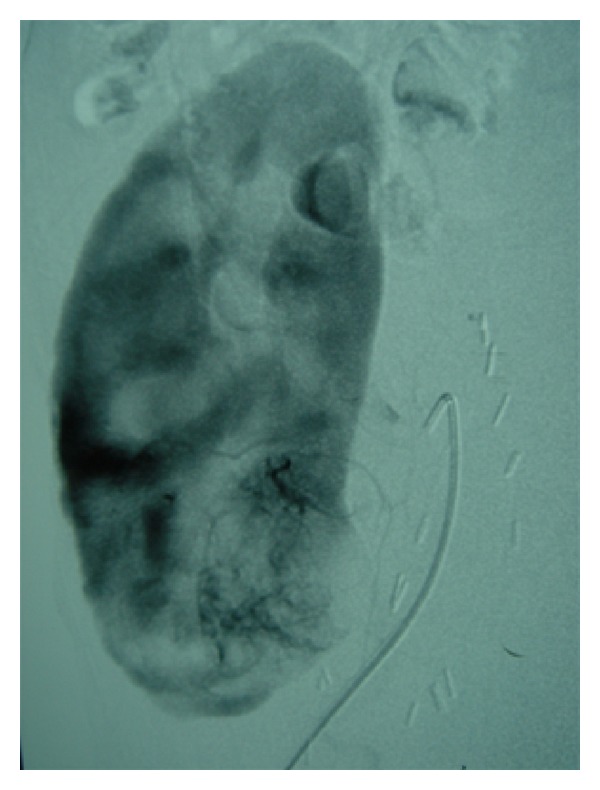
Arteriography showing peripheral tumor in the lower pole of the renal allograft.

We present a 37-year-old woman with previous history of arterial hypertension and terminal chronic renal failure of unknown origin on hemodialysis program for 1 year, who received a cadaveric kidney transplant in the right iliac fossa in 2002. The patient started immunosuppressive treatment with tacrolimus, mycophenolate, and prednisone. Four years after the transplant, a sonographic control revealed a 4.5 cm multilocular cystic mass in the lower pole of the renal allograph that suggested renal cell carcinoma (Figure 1). A subsequent CT scan and arteriography confirmed the diagnosis (Figure 2), and the extension study resulted uneventful. The functioning renal allograph showed basal creatinine levels of 1.7 mg/dL. Because of the size and eccentric location of the tumor, transperitoneal partial nephrectomy of the allograph was performed and ischemia was not necessary. The postoperative course was uneventful. Anatomopathological study revealed a clear-cell renal carcinoma with a 2.5 cm tubular and cystic growth pattern and Furhman nuclear grade II, with no signs of neoplastic infiltration either in peritumoral tissue, hilar or perirenal fat. Six years after surgery, there is no evidence of recurrent disease; renal function is similar to that found before surgery (creatinine 1.7 mg/dL), and modifications in the immunosuppressive treatment were not necessary.

## 3. Discussion

It is well known that patients who undergo solid organ transplant and are under immunosuppressive therapy are in a higher risk of developing cancer. The incidence of malignancies in renal allograft recipients 10 years after transplantation is about 20%, having a 10-time higher risk than the general population. RCC accounts for 4.6% of the tumors in transplant patients; of them, only 10% are found in transplanted kidneys. The Cincinnati Transplant Tumor Registry (CTTR) reported 45 cases of renal cancer of a total of 9,688 cases of cancer developed in 9,032 transplanted patients [2].

RCC in transplanted kidneys is a rare finding that can be due to undetected tumor in the transplanted organ or posttransplantation tumor development [3]. Considering that the growth rate of RCC in native kidneys of transplanted patients is 0.5 cm per year, the lapse of time since transplantation may also provide useful information [4].

Taking into account the relationship between RCC and the immune system, it is possible that tumor inactivity is due to a balance between the good functioning of the immune system and the immune escape mechanisms; this balance is altered by the immunosuppressive agents used in renal transplant [5].

Diagnosis is usually made by sonography studies; CT scan is also useful for diagnosis confirmation and to exclude metastatic dissemination [6].

Fine needle aspiration puncture (FNAP) guided by sonography or CT scan could be an option when images are unclear, although some authors consider this technique a dangerous option with few diagnostic value [7]. Therefore, surgical examination would be recommended in these cases. In our case, renal arteriography was performed in order to determine an adequate management and surgical approach.

Transplantectomy has been traditionally employed, but the quality of life of these patients turns limited again as they have to return to dialysis. However, during the last years, partial nephrectomy is being successfully used [8, 9]. The best conditions to perform nephron-sparing surgery are small size tumors, with an eccentric location, and good blood supply to the rest of the allograft parenchyma [10].

Clinical practice of partial nephrectomy in nontransplanted patients has demonstrated that this technique is a feasible option for localized RCC, with a minimal risk of recurrence or development of metastases and with an acceptable surgical risk [11]; therefore, these indications could be extrapolated to renal transplant patients. Recently, partial nephrectomy of the allograft has also been described using laparoscopic surgery [12].

There is a controversy about the prognostic meaning of positive surgical margin following partial nephrectomy of RCC; however, recent series reported that there is no impact on cancer-specific survival [13, 14]. Nevertheless, conclusive results are difficult to obtain in renal transplant patients with positive surgical margin, since clinical experience is still scarce. In our case, the patient had negative surgical margin.

Sometimes, nephron-sparing surgery can appear technically difficult due to the presence of adhesions from previous surgery. In aged patients with associated comorbidity, alternative techniques to partial surgery could be assessed, such as percutaneous radiofrequency ablation [15] or percutaneous cryoablation [16]; these techniques are simpler, with lower morbidity and shorter hospital stay.

RCC has a better prognosis in transplant kidneys than in native kidneys; partly because of the earlier diagnosis, due to the close followup by imaging studies that is performed in renal transplant patients.

Although RCC is less aggressive in these patients than in the general population, several cases of metastatic spread to different locations have been reported [17].

In conclusion, partial nephrectomy in transplanted kidneys is a feasible option. Renal arteriography may be useful in certain cases in which complicated surgical approach is expected.
